# Identification of reference miRNAs in plasma useful for the study of oestrogen-responsive miRNAs associated with acquired Protein S deficiency in pregnancy

**DOI:** 10.1186/s13104-017-2636-3

**Published:** 2017-07-25

**Authors:** J. W. Tay, I. James, Q. W. Hughes, J. Y. Tiao, R. I. Baker

**Affiliations:** 1 Western Australian Centre for Thrombosis and Haemostasis, Murdoch University, Murdoch, Australia; 2 Perth Blood Institute, Nedlands, Australia; 3 Institute for Immunology and Infectious Diseases, Murdoch University, Murdoch, Australia

## Abstract

**Background:**

Accumulating evidence indicate that circulating microRNAs (miRNAs) are useful independent non-invasive biomarkers, with unique miRNA signatures defined for various pathophysiological conditions. However, there are no established universal housekeeping miRNAs for the normalisation of miRNAs in body fluids. We have previously identified an oestrogen-responsive miRNA, miR-494, in regulating the anticoagulant, Protein S, in HuH-7 liver cells. Moreover, increased thrombotic risk associated with elevated circulating oestrogen levels is frequently observed in pregnant women and oral contraceptive users. In order to identify other oestrogen-responsive miRNAs, including miR-494, that may be indicative of increased thrombotic risk in plasma, we used nanoString analysis to identify robust and stable endogenous reference miRNAs for the study of oestrogen-responsive miRNAs in plasma.

**Results:**

We compared the plasma miRNA expression profile of individuals with: (1) Low circulating oestrogens (healthy men and non-pregnant women not taking oral contraceptives), (2) High circulating synthetic oestrogens, (women taking oral contraceptives) and (3) High circulating natural oestrogens (pregnant females >14 weeks gestation). From the nanoString analyses, 11 candidate reference miRNAs which exhibited high counts and not significantly differentially expressed between groups were selected for validation using realtime quantitative polymerase chain reaction (RT-qPCR) and digital droplet PCR (DDPCR) in pooled plasma samples, and the stability of their expression evaluated using NormFinder and BestKeeper algorithms. Four miRNAs (miR-25-5p, miR-188-5p, miR-222-3p and miR-520f) demonstrated detectable stable expression between groups and were further analysed by RT-qPCR in individual plasma samples, where miR-188-5p and miR-222-3p expression were identified as a stable pair of reference genes. The miRNA reference panel consisting of synthetic spike-ins cel-miR-39 and ath-miR159a, and reference miRNAs, miR-188-5p and miR-222-3p was useful in evaluating fold-change of the pregnancy-associated miRNA, miR-141-3p, between groups.

**Conclusion:**

The miRNA reference panel will be useful for normalising qPCR data comparing miRNA expression between men and women, non-pregnant and pregnant females, and the potential effects of endogenous and synthetic oestrogens on plasma miRNA expression.

**Electronic supplementary material:**

The online version of this article (doi:10.1186/s13104-017-2636-3) contains supplementary material, which is available to authorized users.

## Background

MiRNAs are short, non-coding RNA species approximately 22 nucleotides in length that function to downregulate the expression of its target genes by binding to specific sequences in the 3′ untranslated region (UTR) of target mRNAs leading to the destabilisation of the mRNA or translational repression. Since their discovery in 1993 [1, 2], the role of miRNAs in regulating key cellular processes and their involvement in various pathophysiological conditions, particularly in solid tumours are well studied, and have been extensively reviewed [3–5]. It has also been demonstrated that miRNA profiles for human tumours are superior to mRNA markers, where poorly differentiated tumours could be successfully classified using miRNA expression profiles, whereas mRNA profiles were highly inaccurate when applied to the same tissue samples [6]. A comparison of miRNA and gene expression in frozen breast cancer tissue samples and their paired formalin-fixed paraffin-embedded tissue samples obtained during primary surgical resection showed that expression of miRNAs remains robust even in samples with degraded total RNA, clearly demonstrating the stability of miRNAs in compromised samples in comparison to mRNA markers [7].

The value of miRNAs as useful biomarkers was elevated when plasma and serum were found to be rich in miRNAs that appeared to be protected from RNase degradation [8, 9], and remained highly stable at room temperature and under adverse conditions such as multiple freeze–thaw cycles [10, 11]. A large variety of miRNAs have been detected in various fractions of circulating blood [12] such as erythrocytes [13], anucleate platelets [14], apoptotic bodies [15] and packaged in circulating microparticles shed from platelets and all other cells in the body [16]. As such, we hypothesise that circulating levels of tissue specific miRNAs can provide important information regarding the health status of target tissues, serving as useful non-invasive biomarkers. Due to significant protein and lipid variability between individual plasma and serum samples, a spike-in control is often introduced to correct for variations in RNA extraction efficiency and contamination from inhibitors of PCR (Kroh [33], Sourvinou [47]); although spike-ins have their own caveats. Currently there are no universal endogenous miRNA controls for the study of circulating miRNAs; instead, individual studies arbitrarily designating miRNAs for normalisation.

We demonstrated in HuH-7 liver carcinoma cells that oestrogen treatment resulted in the induction of miR-494 expression in association with downregulated levels of the anticoagulant, Protein S, indicating a mechanism for miRNA-mediated regulation of acquired Protein S deficiency, with a potential role for increased miR-494 levels in pregnant women as an indicator for high thrombotic risk [17]. It is likely that increased circulating oestrogen levels during pregnancy alters the expression of a suite of miRNAs in oestrogen-sensitive tissues, with changes in specific miRNAs possibly detectable in circulating plasma. To this end, we set out to identify stable populations of miRNA which can act as endogenous normalisation markers in human plasma for investigating oestrogen-responsive circulating miRNAs.

## Methods

### Ethics approval

All participants provided written informed consent for the use of venous blood samples in this study prior to blood collection. This project was approved by the Human Research Ethics Committee at Murdoch University (HREC Ref: 2013/131).

### Participants and plasma preparation

Peripheral whole blood was collected from healthy males, non-pregnant females not taking oral contraceptives, non-pregnant females taking oral contraceptives and pregnant females >14 weeks gestation in EDTA Vacutainer^®^ tubes (Becton–Dickinson, NJ, USA). Whole blood was centrifuged at 2000×*g* for 15 min within 4 h of blood collection to separate plasma from cells and platelets as previously described [18], and the supernatant plasma was transferred into RNase-free tubes and stored at −80 °C until required for RNA extraction.

### RNA extraction

Total RNA, including small RNAs was extracted using the miRVANA PARIS RNA extraction kit (Life Technologies, CA, USA), according to the manufacturer’s protocol. For plasma samples, each sample was spiked with 5 μL of 200 nM synthetic cel-miR-39 and ath-miR159a (Sigma-Aldrich, MO, USA) after RNases were inactivated. The extraction protocol was modified with an extended centrifugation time of 60 min at 13,000×*g* at the organic extraction step, and each RNA sample was eluted in 50 μL of hot elution (95 °C) buffer.

### NanoString nCounter^®^ miRNA Assay

miRNA profiling of total RNA extracted from human plasma samples was performed using the nCounter^®^ Human miRNA Panel v2 (NanoString, Seattle, USA). Plasma total RNA samples were concentrated using a 3 kDa size exclusion filter (Sigma-Aldrich, MO, USA) and 3 μL of concentrated total RNA was hybridised to probes, purified and counted on the nCounter^®^ Prep Station and Digital Analyser according to the manufacturer’s instructions. For each sample, 555 fields of view (FOV) were imaged and counted by the nCounter^®^ Digital Analyser.

### Availability of data and materials

All supporting data for all samples analysed by NanoString nCounter^®^ miRNA Assay is presented in this article or as Additional file 1.

### Reverse transcription and miRNA expression studies by real-time PCR (RT-PCR) and droplet digital PCR (DDPCR)

Total RNA samples were reverse transcribed using the TaqMan^®^ microRNA reverse transcription kit (Life Technologies, CA, USA), and selected mature miRNAs were detected using TaqMan^®^ miRNA assays (Table 1) (Life Technologies, CA, USA) on the CFX96/384 real-time PCR system (Bio-Rad, CA, USA). Absolute quantitation of miRNA concentration in plasma samples was directly determined using the same TaqMan^®^ miRNA assays and droplet digital PCR (DDPCR) (Bio-Rad, CA, USA). The miRNA concentrations (copies/μL plasma) were calculated using the formula: (copy number of miRNA per microlitre of DDPCR reaction mixture) × (the dilution factor in the plasma with RNA extraction, reverse transcription and DDPCR reaction), and finally normalised to the concentration of cel-miR-39 and ath-miR159a.

**Table 1:** Mean and standard deviation of nanostring counts of candidate housekeeping miRNAs in order of decreasing average counts

Candidate housekeeping miRNA	miRBase accession number	Mature miRNA sequence	TaqMan assay ID	Nanostring raw counts			
				Male	Non-pregnant	Oral contraceptive	Pregnant
				n = 4	n = 4	n = 4	n = 4
				Age: 37 ± 14	Age: 34 ± 15	Age: 30 ± 3	Age: 29 ± 5
miR-25-3p	MIMAT0000081	CAUUGCACUUGUCUCGGUCUGA	000403	232 ± 64	211 ± 22	225 ± 31	278 ± 77
miR-520f	MIMAT0002830	AAGUGCUUCCUUUUAGAGGGUU	001120	109 ± 25	101 ± 16	125 ± 33	89 ± 5
miR-149-5p	MIMAT0000450	UCUGGCUCCGUGUCUUCACUCCC	002255	85 ± 13	82 ± 16	80 ± 6	72 ± 13
let-7a-5p	MIMAT0000067	UGAGGUAGUAGAUUGUAUAGUU	000377	76 ± 17	87 ± 15	79 ± 32	80 ± 16
miR-2682-5p	MIMAT0013517	CAGGCAGUGACUGUUCAGACGUC	463610_mat	74 ± 8	71 ± 10	74 ± 14	75 ± 2
miR-612	MIMAT0003280	GCUGGGCAGGGCUUCUGAGCUCCUU	001579	61 ± 9	70 ± 9	70 ± 9	60 ± 11
miR-222-3p	MIMAT0000279	AGCUACAUCUGGCUACUGGGU	002276	51 ± 15	52 ± 7	62 ± 9	53 ± 4
miR-598	MIMAT0003266	UACGUCAUCGUUGUCAUCGUCA	001988	46 ± 10	62 ± 3	60 ± 11	58 ± 8
miR-188-5p	MIMAT0000457	CAUCCCUUGCAUGGUGGAGGG	002320	50 ± 8	61 ± 2	48 ± 12	55 ± 3
miR-489	MIMAT0002805	GUGACAUCACAUAUACGGCAGC	002358	49 ± 16	55 ± 12	49 ± 12	46 ± 8
miR-1183	MIMAT0005828	CACUGUAGGUGAUGGUGAGAGUGGGCA	002841	46 ± 6	58 ± 4	54 ± 18	41 ± 12

### Statistical analysis and evaluation of candidate reference miRNA stability

The raw counts obtained from the nCounter^®^ Digital Analyser were compared overall among the four cohorts and via pairwise cohort comparisons based on quasi-likelihood regression with a log link and variance proportional to the square of the mean [19], which upon inspection indicated was an appropriate model. The regressions adjusted simultaneously for the sum of the logs of the internal positive controls and the synthetic spike-in miRNA, ath-miR159a. Candidate housekeeping miRNAs were identified if they had raw counts >40 and were not significantly different between samples analysed (p > 0.05). Analyses were carried out in TIBCO Spotfire S+ ver 8.2 (Boston, MA).

The stability of miRNA expression was evaluated using NormFinder [20] and Bestkeeper [21] software with averaged Cq values to select the most stable reference miRNAs. The 2^−∆∆Ct^ method was used to calculate the expression of the target miRNAs relative to suitable reference miRNAs, including the two synthetic spike-in miRNAs, cel-miR-39 and ath-miR159a.

## Results

### Identification of candidate housekeeping miRNAs by nanoString nCounter^®^ miRNA assay

**Fig. 1:**
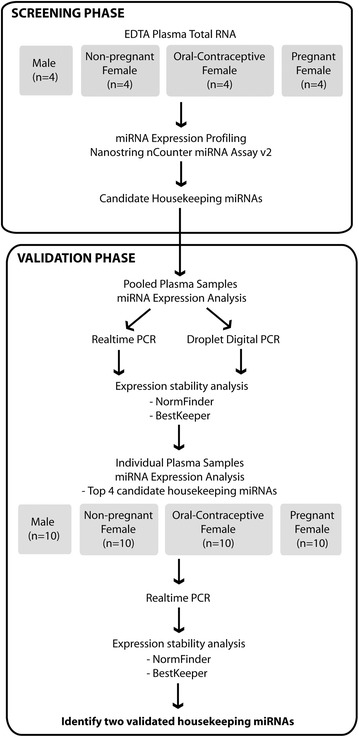
Schematic of workflow for identifying stable housekeeping miRNAs in this study

In order to identify suitable candidate housekeeping miRNAs for studying oestrogen-responsive miRNAs in plasma, the expression profiles of 800 miRNAs in human plasma samples collected from healthy males, non-pregnant females not taking oral contraceptives, non-pregnant females currently taking oral contraceptives and pregnant females (>14 weeks gestation) were obtained using the nanoString nCounter^®^ miRNA assay v2, which comprised the screening phase (Fig. 1). The candidate housekeeping miRNAs were then validated using RT-qPCR and DDPCR for oestrogen-non-responsiveness between the cohorts in total RNA extracted from pooled plasma samples and the miRNAs identified as maintaining robust expression and concordance with nanoString were further analysed in individual plasma samples by RT-qPCR (Fig. 1).

Out of the 800 miRNAs analysed using the nanoString nCounter^®^ miRNA assay v2, 760 miRNAs had average counts less than 46, of which ~600 miRNAs showed average nanoString counts of less than 20. As such, the initial selection criterion for classifying detectable miRNAs at the screening phase was set at 40 counts to include miRNAs that were considered “low positives”. The results showed that 44 out of 800 miRNAs analysed showed average nanoString counts greater than 40 in all 16 plasma samples, of which the counts for 11 miRNAs were not significantly different by pairwise comparisons (*p* > 0.05) and identified as suitable candidate reference miRNAs for further analysis (Table 1). Of the 11 candidate miRNAs, miR-25-3p was the most abundant miRNA in the plasma samples analysed, followed by miR-520f, miR-149-5p, let-7a-5p, miR-2682-5p, miR-612, miR-222-3p, miR-598, miR-188-5p, miR-489 and lastly miR-1183 exhibiting the lowest average nanoString counts (Table 1).

### Validation of candidate housekeeping miRNAs by qPCR and DDPCR in pooled and individual plasma samples

**Fig. 2:**
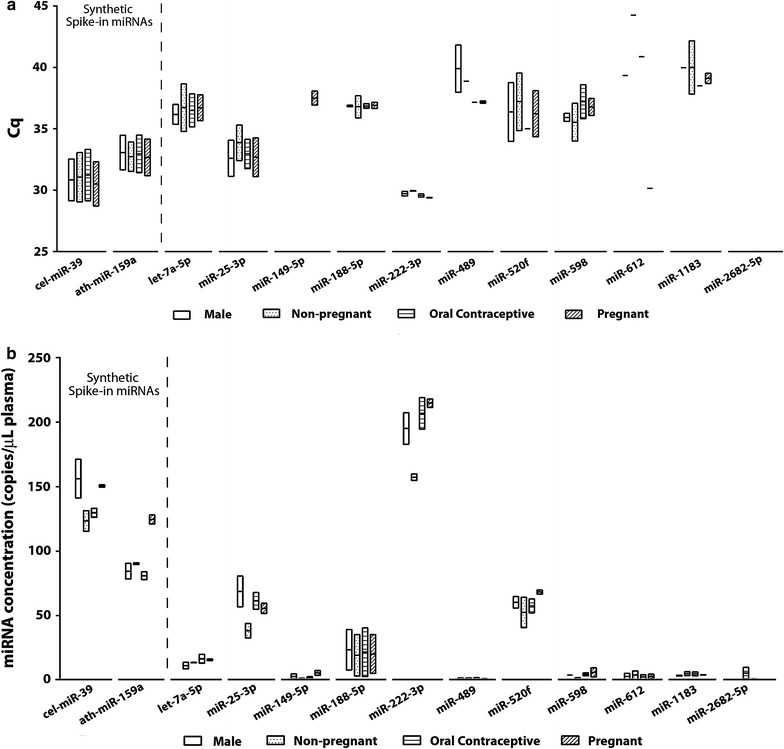
Evaluation of candidate miRNA expression levels in pooled plasma samples from healthy males, non-pregnant females not on oral contraceptives, non-pregnant females taking oral contraceptives and pregnant females determined by **a** real-time PCR or **b** digital droplet PCR. Results were analysed from two technical replicates presented as *box plots* with mean and 1 standard deviation

To validate the stability of the selected candidate housekeeping miRNAs, equal volumes of plasma samples collected were pooled within each cohort and total RNA was extracted from the pooled plasma, reverse transcribed and analysed by qPCR and DDPCR, the whole process performed twice as technical replicates. There was poor concordance of miRNA expression between nanoString and qPCR results, miRNAs which showed high counts in nanoString, miR-149-5p, miR-489, miR-612, miR-1183 and miR-2682 were not amplified under 40 cycles using qPCR TaqMan^®^ microRNA assays. This observation was matched with low miRNA concentrations using DDPCR methods (Fig. 2).

Based on Cq values, miR-222-3p exhibited the highest relative abundance, with an average Cq of 29, followed by miR-25-3p, miR-520f, let-7a-5p and miR-188-5p (Fig. 2a), where the order of abundance was matched to the corresponding miRNA concentrations determined by DDPCR (Fig. 2b). MiR-598 was an exception, however, where its absolute counts determined by DDPCR were markedly lower than expected (<5copies/μL plasma), in contrast to its relative expression levels determined by RT-qPCR with an average Cq of 35 (Fig. 2). The stability scores of the candidate miRNAs were determined using NormFinder and Bestkeeper which identified miR-25-3p, miR-188-5p, miR-222-3p and miR-520f as the miRNAs that exhibited low variability across the four groups analysed (Table 2), as well as high expression determined by qPCR and DDPCR. Although let-7a-5p was ranked the 4th most stable miRNA, with miR-25-3p being 5th, the expression levels of miR-25-3p were markedly higher than that of let-7a-5p expression levels in nanoString, qPCR and DDPCR analyses, in association with good stability values for miR-25-3p, as determined by NormFinder and Bestkeeper. Consistent and robust detection across platforms is the top selection criteria; therefore, miR-25-3p, miR-188-5p, miR-222-3p and miR-520f were selected for further analyses in individual plasma samples.

**Table 2:** Ranking of candidate reference genes in pooled plasma samples based on stability values calculated by NormFinder and BestKeeper

Rank	NormFinder		BestKeeper	
	miRNA	Stability value	miRNA	Stability value
1	miR-188-5p	0.102	miR-222-3p	0.19
2	miR-222-3p	0.487	miR-188-5p	0.35
3	miR-598	1.034	miR-598	1.07
4	let-7a-5p	1.280	let-7a-5p	1.33
5	miR-25-3p	1.323	miR-25-3p	1.44
6	miR-149-5p	2.232	miR-520f	1.79
7	miR-520f	4.592	miR-489	1.88
8	miR-489	7.030	miR-612	8.74
9	miR-1183	7.345	miR-149-5p	9.54
10	miR-612	11.463	miR-1183	–
11	miR-2682-5p	–	miR-2682-5p	–

**Fig. 3:**
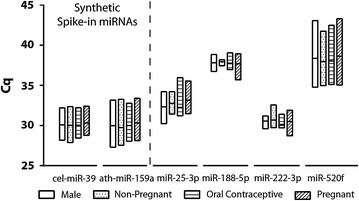
Cq values of candidate housekeeping miRNAs in individual plasma samples (n = 10 per group). Results presented as *box plots* with mean and 1 standard deviation

The expression levels of the four candidate miRNAs were analysed by qPCR in 40 individual plasma samples (10 plasma samples per group) and the variability of each miRNA across the plasma samples analysed determined by the stability scores calculated using NormFinder and BestKeeper. The relative abundance, determined by RT-qPCR, of miR-222-3p was the most highly expressed in all plasma samples with the lowest average Cq, followed by miR-25-3p, then miR-188-5p and lastly miR-520f (Fig. 3). These results were in agreement with that obtained using pooled plasma samples where the selected miRNAs exhibited the same order of abundance.

Stability analysis of the four candidate miRNAs in individual plasma samples using NormFinder generated stability scores of 0.115–0.601, indicating that all four miRNAs were good reference miRNAs, with miR-25-3p, miR-188-5p and miR-222-3p being the top three least variable miRNAs (in order of increasing variability) in the individual plasma samples tested (Table 3). Bestkeeper analysis showed that miR-188-5p was the most stable miRNA, followed by miR-222-3p and miR-25-3p, where the stability score for miR-25-3p was 1.30 and not in agreement with the level of variability determined by the NormFinder algorithm. As the stability analysis for miR-188-5p and miR-222-3p were consistent between NormFinder and Bestkeeper with stability scores in both cases indicating low variability, miR-188-5p and miR-222-3p were chosen as the two endogenous reference miRNAs for plasma miRNA quantitation.

**Table 3:** Normfinder and BestKeeper analyses of candidate housekeeping miRNAs in individual plasma samples

Rank	NormFinder		BestKeeper	
	miRNA	Stability value	miRNA	Stability value
1	miR-25-3p	0.115	miR-188-5p	0.55
2	miR-222-3p	0.175	miR-222-3p	0.58
3	miR-188-5p	0.345	miR-25-3p	1.30
4	miR-520f	0.601	miR-520f	3.10

### Reference housekeeping panel is effective for normalising RT-qPCR data for circulating miRNAs

Many oestrogen-responsive miRNAs have been identified, however the studies were performed in breast cancer cell lines and other cell types [22–24]. The effects of high oestrogen levels on miRNAs in circulation have not been investigated, and there is also little information regarding gender associated miRNA expression profiles. As such, in order to validate the housekeeping miRNA panel, we determined the relative expression levels of a pregnancy-associated miRNA, miR-141-3p in total RNA extracted from individual plasma samples from each group using RT-qPCR. Levels of miR-141-3p have been reported in maternal serum and plasma samples to be increased with gestational age [11, 25], and significantly decreased following termination of pregnancy [25, 26]. The raw counts for miR-141-3p obtained from nanoString nCounter^®^ analysis here ranged from 15 to 35 counts in the samples analysed and did not show significant difference between the groups by pairwise comparisons.

**Fig. 4:**
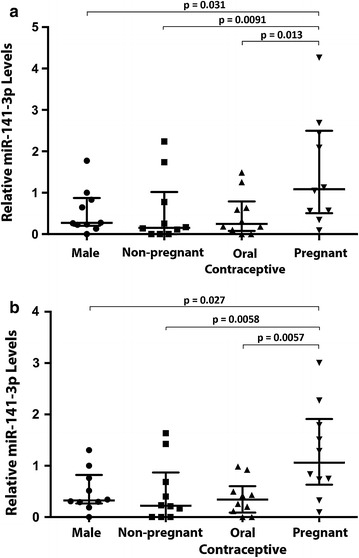
Normalisation of the relative expression of pregnancy-associated miRNA, miR-141-3p with the miRNA reference panel increases the significance of group differences compared to normalisation with spike-ins alone. **a** miR-141-3p levels normalised to synthetic spike-ins cel-miR-39 and ath-miR159a alone. **b** miR-141-3p levels normalised to synthetic spike-ins and endogenous controls miR-188-5p and miR-222-3p. *Horizontal bars* represent median with interquartile range. Statistical significance determined by one way ANOVA based on the square root transformation of the relative expression levels. Overall significance for differences between the four groups **a** p = 0.031, **b** p = 0.0166

The relative expression of miR-141-3p in individual plasma samples between the four groups was analysed using RT-qPCR with various normalisation strategies (Fig. 4). When normalised to spike-ins alone, levels of miR-141-3p were significantly higher in pregnant samples compared to males, non-pregnant and oral contraceptive (p < 0.05) as expected (Fig. 4a). Overall there was a significant difference between the means in the four groups (p = 0.031). Individually, miR-141-3p expression in each cohort was lower than the pregnant group, namely healthy males (p = 0.031), oral contraceptives (p = 0.013) and non-pregnant women (p = 0.0091). There was no significant difference between the pairwise comparisons of miR-141-3p expression between healthy males, non-pregnant females not on oral contraceptives and non-pregnant females taking oral contraceptives with pregnant females (p = 0.87). In addition, as a group miR-141-3p expression was also significantly lower than that of the pregnant group (p = 0.0030).

When the RT-qPCR data was normalised to both the synthetic spike-ins and endogenous miRNA controls miR-188-5p and miR-222-3p, the relative levels of miR-141-3p was also significantly increased in pregnant samples compared to non-pregnant and oral contraceptive samples (*p* < 0.05), similar to results obtained when the data was normalised to spike-ins alone. The significance levels between cohorts, however, increased with the inclusion of the endogenous reference miRNAs in the normalisation (Fig. 4b). Overall there was a significant difference between the means in the four groups (p = 0.0166). Individually, miR-141-3p expression in each cohort is lower than that in the pregnant group, namely healthy males (p = 0.027), oral contraceptive women (p = 0.0057) and non-pregnant women (p = 0.0058). There was no significant difference in miR-141-3p expression between the healthy male, oral contraceptive women and non-pregnant women groups (p = 0.77), but as a group, their miR-141-3p levels were significantly lower than the pregnant women group (p = 0.0016).

## Discussion

Mounting evidence highlight the value of circulating miRNAs as a source of stable non-invasive biomarkers, with unique circulating microRNA profiles defined for various pathological conditions [27–31]. However, the lack of universal housekeeping controls and standardised methods for quantitating circulating miRNAs remains a major hurdle for the accurate analysis of miRNA expression in biofluids. This caveat also hinders translation of disease-associated miRNA signatures characterised using high throughput methods such as microarrays and next generation sequencing methods into diagnostic tests in the laboratory. As qPCR-based methods are relatively affordable and accessible for most laboratories, it remains the gold standard for miRNA analysis. Therefore, the identification of a robust endogenous miRNA control(s) specific for each condition with stable and consistent level of expression across platforms is fundamentally important.

Precedence exists for utilising short RNAs as endogenous gene normalisation factor(s), albeit with certain pitfalls. Small nucleolar RNAs (snoRNA) such as RNU6B, which is highly expressed in cells [32], and miR-16 which is abundantly expressed in blood are frequently used normalisation factors for miRNA studies [33–36], but their suitability as endogenous controls is controversial. RNU6B is present at low concentrations or shown to exhibit wide concentration variations in circulation [37], and was reported to be less resistant to multiple freeze–thaw cycles compared with other mature miRNA species analysed in serum samples [38]. Red blood cells contain high levels of miR-16 [39], and studies have demonstrated that expression of miR-16 was significantly increased in haemolysed plasma samples, with the miR-16 levels proportional to the degree of haemolysis [40, 41], indicating that miR-16 is more useful as a haemolysis marker than a normalisation factors. Furthermore, miR-16 has been reported to be responsive to steroid hormones, downregulated by progestin and oestradiol in breast cancer cells [42, 43], and serum miR-16 is part of a diagnostic/prognostic miRNA panel for ovarian cancer patients [44]. Taken together, there exists a need for a stable and non-hormone responsive miRNA in normalising endogenous miRNA expression levels in human plasma.

As an initial screen of 11 candidate reference miRNAs selected from the nanoString nCounter^®^ assay, we analysed the expression of those candidate miRNAs in pooled plasma samples of (1) males, (2) non-pregnant females not on oral contraceptives, (3) non-pregnant oral contraceptive users and (4) pregnant females. Out of the 11 candidate reference miRNAs, 5 of them were detected at low levels (Cq > 38) or not amplified by qPCR or DDPCR, demonstrating marked discordance between the detection methods. This observation highlighted platform variations between nanoString and qPCR-based methods. The results reported here showed that many of the candidate miRNAs which exhibited high counts in nanoString did not correspondingly demonstrate high abundance when analysed using RT-qPCR and DDPCR, and low nanoString counts may not necessarily mean poor detectability via PCR amplification methods. nCounter^®^ is a hybridisation based technology with high specificity without an amplification step; eliminating amplification bias introduced in qPCR methods. Low sensitivity, however, is a major limitation of nanoString, especially for the analysis of low input RNA samples such as serum/plasma RNA, which can be overcome by qPCR techniques. As such, it may not be surprising that the low concordance between nanoString and qPCR-based results observed in this present study has also been reported by other groups looking at circulating miRNAs. A study evaluating the performance of microRNA array platforms using different breast cancer cell lines found that hybridisation-based technology such as the nCounter^®^ system demonstrated lower sensitivity compared to next generation sequencing and PCR-based platforms [45]. Mestdagh et al. [46] conducted a large scale analysis of commercially available quantitative miRNA expression profiling platforms and evaluated them based on reproducibility, sensitivity, accuracy, specificity and concordance of differential expression. Their results demonstrated substantial inter-platform differences associated with each type of technology coupled with an inverse correlation between sensitivity and specificity [46]. In this present study, the nanoString nCounter^®^ miRNA assay v2 was useful as an initial screening tool to identify highly expressed miRNAs but users should be aware of false negatives due to the reduced assay sensitivity. Regardless, the results obtained in this study using nanoString led to the identification of miR-188-5p and miR-222-3p from human plasma samples, which exhibited consistently stable levels of expression between the cohorts analysed, across two measurement platforms.

During RNA extraction, there is significant protein and lipid variability between individual plasma or serum samples, which may affect RNA extraction efficiency and introduce potential PCR inhibitors [33, 47], therefore, a synthetic spike-in control is necessary. However, synthetic spike-in miRNAs only correct for differences in extraction efficiency and are not useful in normalising endogenous miRNA content. This may be sufficient for studies that have standardised pre-analytical conditions including collection, plasma preparation, sample storage and total RNA extraction methods. Hence it is prudent that suitable reference miRNAs are established for each study with appropriate test samples to avoid introducing bias in the analysis and skewing the final results. In this study, we used the nanoString nCounter^®^ miRNA assay to obtain the plasma miRNA profiles of non-pregnant women, women who are currently taking oral contraceptives and pregnant females, mixed groups that represent individuals with low and high circulating oestradiol concentrations, respectively. From this data, we identified two stable endogenous reference miRNAs that are suitable for the normalisation of plasma miRNA expression in addition to synthetic spike in miRNAs, such as cel-miR-39 and ath-miR159a, which will allow for more accurate qPCR normalisation for studying oestrogen responsive miRNAs that may be involved in regulating other haemostatic factors leading to Protein S deficiency during pregnancy.

The independent plasma analyses of four candidate miRNAs, miR-25-3p, miR-188-5p, miR-222-5p and miR-520f showed that miR-520f expression exhibited high inter-individual variability, with Cq > 38 for many samples tested. The stability analyses using NormFinder and Bestkeeper showed that the remaining three miRNAs were stable between individual plasma samples tested but based on the stability scores, miR-188-5p and miR-222-3p were selected as the reference miRNAs. Both miR-188-5p and miR-222-3p have been previously evaluated as housekeeping miRNAs in plasma samples from cancer patients [48] and in breast tumour tissue biopsies [49], respectively, but were not ultimately chosen as reference miRNAs due to variability between the cancer cohorts. This indicates that miR-188-5p and miR-222-3p were not suitable for cancer studies, but it validates the data in this study that miR-188-5p and miR-222-3p exhibited and robust expression in multiple tissue types.

Furthermore, it should be noted that while miR-188-5p and miR-222-3p were found to be stable in the cohorts analysed in this study, they do have ascribed biological functions. In hepatocellular carcinoma cells, miR-188-5p was shown to inhibit growth of hepatocellular carcinoma cells by targeting the fibroblast growth factor [50] and was also demonstrated to be growth inhibitory in prostate cancer cells [51]. In contrast, miR-222-3p has been reported to be important in the epithelial mesenchymal transition in breast cancer cell lines and its expression associated with ERK1/2 signalling activity [52]. Expression of miR-222-3p has also been shown to be upregulated by selective estrogen receptor downregulator (SERD), fulvestrant in the breast cancer cell line, MCF-7 [53]. In circulation, Vickers et al. [54] have found miR-188-5p to be highly abundant in high density lipoproteins and circulating levels of miR-222-3p have been demonstrated to be a useful diagnostic marker in gastric cancer; and miR-222-3p has been reported to negatively regulate the expression of ERα in endometrial carcinoma cells [55], but oestrogen-mediated regulation of miR-222-3p and miR-188-5p have not been previously reported.

RT-qPCR analyses of miR-141-3p expression was chosen to adjudicate the novel miRNA normalisation panel established in this study. Levels of serum and plasma miR-141-3p have been reported by multiple studies to be significantly upregulated in serum and plasma samples of pregnant women using PCR-based miRNA arrays [11, 56], and as such, serves as an ideal candidate for validating the reference normalisation panel. The results presented here showed that significant changes in miR-141-3p expression between non-pregnant (with or without oral contraceptives) and pregnant females can be observed when the RT-qPCR data was normalised to the synthetic spike-ins. This is consistent with previously published data, despite a different normalisation strategy being applied in each study. Here, we demonstrated that greater levels of significance in the differences in expression levels were observed when miR-188-5p and miR-222-3p were introduced into the normalisation panel with the spike-ins, compared to normalisation with the spike-ins alone. Importantly, this suggests that small, but significant differences in miRNA expression initially overlooked when results were normalised to synthetic spike-ins alone will be identified by including endogenous miRNA normalisation factor(s). Circulating miR-141-3p has been previously investigated as a marker for metastatic disease in prostate cancer patients [57] and also in colon cancer patients [58]. A more recent study has identified miR-141-3p to be a novel biomarker for metastatic colorectal cancer in plasma samples from male and female patients [58, 59]. However, miR-141-3p levels were either only determined in men or there was not a comparison of relative miR-141-3p expression between male and female patient cohorts in these studies.

To date, there are no reports describing reference miRNAs for the analysis of hormone-responsive miRNAs in human serum and plasma samples. Furthermore, studies investigating oestrogen-responsive miRNAs have predominantly been performed in human breast cancer cell lines [22, 23, 60, 61]; whereas, hormone induced miRNAs in thrombosis remains poorly characterised. Thus the accurate identification of miRNAs regulated during pregnancy under high levels of circulating oestrogen is critical in assessing which target haemostatic genes could heighten thrombotic risks. It is vital, therefore, that suitable endogenous miRNAs are established in the appropriate study cohorts. In addition, there have been many studies which characterised miRNA expression profiles associated with pregnancy complications such as preeclampsia [62, 63], ectopic pregnancies [56] and gestational diabetes [64]. Baseline levels, however, are absent as these studies compared expression profiles between pregnant women only and not against non-pregnant participants. This current study is the first report for the systematic identification of reference miRNAs for the normalisation of miRNA qPCR data in studies investigating oestrogen-responsive miRNAs.

While the miRNA reference panel reported in this study has been established for identifying novel miRNAs that contribute to increased thrombotic risk in pregnant women with high circulating oestrogen levels compared to non-pregnant women and women taking oral contraceptives, it can be applied in a number of other biological contexts. The control miRNAs were validated to be stable between healthy men, non-pregnant women, women on oral contraceptive and pregnant women. As such, it may be used to normalise miRNA expression in studies investigating gender-specific associations of miRNA expression in disease. A recent study conducted a comprehensive comparison of miRNA expression profiles in healthy and tumour tissues between men and women demonstrated marked gender associated differences in miRNA/isomiR expression [65]. Reports of associations of gender in a population based study [66] and influence of gender differences on microRNA gene regulation in disease [67] all support the need for the identification of gender neutral miRNAs that can be used in studies that compare miRNA expression between male and female patient cohorts. The miRNA reference panel in this report will also be useful in the comparison of plasma miRNA expression regulated by synthetic and natural oestrogens in healthy individuals, with the caveat that the stability of the reference miRNAs requires validation in breast cancer patients or women with polycystic ovarian syndrome. Furthermore, the less than ideal inter-platform comparability of miRNA expression between currently available commercial platforms reported by multiple studies should be taken into account [45, 46, 68, 69]. As such, the strategy for establishing suitable reference miRNAs in any biological context should employ a different detection platform for screening and validation, i.e. reverse transcription quantitative PCR versus hybridisation.

## Conclusions

Endogenous controls for circulating miRNAs are not universal and the choice of normalising miRNAs must be carefully selected for each study. If a previously reported endogenous control miRNA is absent, it is pertinent that suitable housekeeping miRNAs be established in the study cohorts using our reported strategy. Also, the variability in lipid content of biological fluids between individuals can affect RNA extraction efficiencies, as such, the addition of synthetic spike-ins cel-miR-39 and ath-miR159a are necessary as first line controls for normalising variations in extraction efficiency; however, synthetic spike-ins alone, are not adequate for normalisation. The results presented here identified two plasma miRNAs, miR-188-5p and miR-222-3p, whose expression levels were stable between the four cohorts analysed, representing individuals with low and high circulating endogenous oestradiol concentrations, as well as high circulating synthetic oestrogens, and should be included in the normalisation panel for the identification of oestrogen responsive miRNAs in circulation. The four reference miRNA panel established in this study may also be useful for normalising qPCR data comparing miRNA expression between men and women, non-pregnant and pregnant females, and also the potential effects of endogenous and synthetic oestrogens on plasma miRNA expression.

## Additional file

**Additional file 1.** Nanostring raw data. Nanostring raw counts for platelet poor plasma samples and pairwise comparisons between groups.

## Abbreviations

3′UTR: 3 prime untranslated region
miRNA: microRNA
RT-qPCR: real time quantitative polymerase chain reaction
